# Adjusting for measurement error in baseline prognostic biomarkers included in a time-to-event analysis: a joint modelling approach

**DOI:** 10.1186/1471-2288-13-146

**Published:** 2013-12-01

**Authors:** Michael J Crowther, Paul C Lambert, Keith R Abrams

**Affiliations:** 1 University of Leicester, Department of Health Sciences, Adrian Building, University Road, Leicester LE1 7RH, UK; 2 Karolinska Institutet, Department of Medical Epidemiology and Biostatistics, Box 281, S-171 77 Stockholm, Sweden

## Abstract

**Background:**

Methodological development of joint models of longitudinal and survival data has been rapid in recent years; however, their full potential in applied settings are yet to be fully explored. We describe a novel use of a specific association structure, linking the two component models through the subject specific intercept, and thus extend joint models to account for measurement error in a biomarker, even when only the baseline value of the biomarker is of interest. This is a common occurrence in registry data sources, where often repeated measurements exist but are simply ignored.

**Methods:**

The proposed specification is evaluated through simulation and applied to data from the General Practice Research Database, investigating the association between baseline Systolic Blood Pressure (SBP) and the time-to-stroke in a cohort of obese patients with type 2 diabetes mellitus.

**Results:**

By directly modelling the longitudinal component we reduce bias in the hazard ratio for the effect of baseline SBP on the time-to-stroke, showing the large potential to improve on previous prognostic models which use only observed baseline biomarker values.

**Conclusions:**

The joint modelling of longitudinal and survival data is a valid approach to account for measurement error in the analysis of a repeatedly measured biomarker and a time-to-event. User friendly Stata software is provided.

## Background

Many biomarkers such as systolic blood pressure (SBP) have been identified as key prognostic factors in the development and validation of cardiovascular risk scores [1,2]. However, often only baseline values of these biomarkers are used, despite the existence of repeated measures, especially in registry sources such as the General Practice Research Database (GPRD) [3]. Furthermore, biomarkers are often measured with error. Failing to adjust for such measurement error leads to estimates being biased towards the null [4].

A joint model of longitudinal and survival data allows us to investigate the relationship between a repeatedly measured biomarker, subject to measurement error, such as SBP, and the time to an event of interest, such as time to non-fatal stroke. The approach which has dominated the methodological literature involves linking the two component submodels using shared random effects [5,6]. From a classical perspective, these methods require computationally intensive numerical integration, which is difficult to implement. However, due to the recent introduction of user-friendly software in R [7,8] and Stata [9], these models are starting to find their place in applied research [10,11], but the potential uses of and forms of the association parameters, linking the longitudinal and survival components, are yet to be fully explored. Alternatively, many authors have proposed a Bayesian approach, proving readily available BUGS code to implement the models [12,13].

The most commonly used association structures include the current value parameterisation [5]; whereby we directly link the value of the biomarker, as estimated by the longitudinal submodel, to survival, and the first derivative or slope [10]; allowing the investigation of the effect that the rate of change of the biomarker has on survival.

There is often interest in predicting prognosis based on an initial baseline measurement [1,2]. In this paper we investigate the use of the joint model framework with a random intercept association structure as an approach to adjust for measurement error, inherent in biomarkers such as SBP. By incorporating the repeated measures we thus make the most efficient use of the data available. In particular, as a prognostic model for future patients, we describe how this framework can be used to predict survival for new patients who will only have baseline measurements.

## Methods

A joint model of longitudinal and survival data consists of two component submodels: the longitudinal submodel and the survival submodel. We define a set of baseline covariates, ***U***_*i*_, which can potentially differ between submodels. The longitudinal submodel allows us to model the trajectory of a repeatedly measured biomarker over time, adjusting for baseline covariates. The standard approach assumes a linear mixed effects model [14]. We observe

(1) $$Y_{i}(t_{\text{ij}})=W_{i}(t_{\text{ij}})+\varepsilon_{\text{ij}},\;\varepsilon_{\text{ij}}∼N(0,\sigma_{e}^{2})$$

with

(2) $$W_{i}(t_{\text{ij}})=X_{i}^{\prime }(t_{\text{ij}})\beta +Z_{i}^{\prime }(t_{\text{ij}})b_{i}+u_{i}\delta$$

where *Y*_*i*_(*t*_*ij*_) is the observed longitudinal response for the *i*^*th*^ patient measured at the *j*^*th*^ time point. *W*_*i*_(*t*_*ij*_) is our true unobserved trajectory function consisting of design matrices $X_{i}^{\prime }(t_{\text{ij}})$ and $Z_{i}^{\prime }(t_{\text{ij}})$ for the fixed and random effects, ***β*** and ***b***_*i*_, respectively, where ***b***_*i*_ ∼ MVN(0,**Σ**). We can incorporate flexibility here by allowing both $X_{i}^{\prime }(t_{\text{ij}})$ and $Z_{i}^{\prime }(t_{\text{ij}})$ to contain restricted cubic spline functions of measurement time [15]. We also have a vector of baseline covariates ***u***_*i*_ ∈ ***U***_*i*_, and corresponding regression coefficients, ***δ***. Finally, *ε*_*ij*_ is our normally distributed measurement error with constant variance $\sigma_{e}^{2}$. We further assume that the random effects and error term are independent, and that cov (*ε*_*ij*_,*ε*_*ik*_) = 0 (where *j* ≠ *k*).

The time-to-event submodel usually takes the form of a proportional hazards model

(3) $$h_{i}(t)=h_{0}(t)\exp(\alpha_{1}W_{i}(t)+\phi v_{i})$$

with *h*_0_(*t*) the baseline hazard function and ***v***_*i*_ ∈ ***U***_*i*_ is a vector of baseline covariates with corresponding log hazard ratios, ***ϕ***. The parameter *α*_1_ is commonly named the association parameter, indicating the strength of association between the longitudinal biomarker and the time to event. If *α*_1_ = 0, then the joint model reduces to the two separate models and fitting a joint model will not prove advantageous. This parameterisation assumes the hazard is dependent on the biomarker through its current value. This form of association is one of many ways to link the two component sub-models. The baseline hazard function, *h*_0_(*t*), can be modelled using a parametric distribution, most frequently the Weibull, or less restrictively using flexible parametric survival models [16], or of course can be left unspecified [17]. However, an unspecified baseline hazard function leads to underestimation of the standard errors of parameter estimates [18], and consequently bootstrapping is required to obtain appropriate standard errors.

For illustration, we let *W*_*i*_(*t*_*ij*_), the longitudinal submodel, be a linear function of time where the intercept and slope varies between subjects

(4) $$W_{i}(t_{\text{ij}})=(\beta_{0}+b_{0i})+(\beta_{1}+b_{1i})t_{\text{ij}}$$

giving a model with a random intercept and random linear slope. As an alternative way of linking the component models to that of Equation (3), we may link elements of the trajectory function, *W*_*i*_(*t*_*ij*_), to the hazard directly. For example, we can link the subject specific baseline biomarker values through the intercept association structure, where

(5) $$h_{i}(t)=h_{0}(t)\exp\left[\alpha_{2}(\beta_{0}+b_{0i})+\phi v_{i}\right]$$

in this expression *α*_2_ now estimates the strength of the association between the patient specific baseline biomarker values, as estimated by the longitudinal submodel, and the time-to-event. This way we can let the risk of event depend directly on the subject specific value of the biomarker at time *t* = 0.

If interest lies in prediction when a new patient is observed at baseline, the issue of measurement error can be accounted for through this approach. A benefit of this association structure also lies in the evaluation of the joint likelihood. Under most parametric survival submodels (e.g. Weibull distribution) and time-dependent association structures (eg. current value), numerical quadrature is required to integrate out not only the random effects, but under Equation (3), nested quadrature is also required to evaluate the cumulative hazard function. Under the time-independent association structure of Equation (5), we avoid this nested quadrature as the cumulative hazard function has an analytically tractable form, which provides computational benefits.

As discussed in the introduction, this model formulation can be an alternative to the standard approach of using the observed baseline biomarker value

(6) $$h_{i}(t)=h_{0i}(t)\exp(\alpha_{3}Y_{0i}+\phi v_{i})$$

where *Y*_0*i*_ is the observed baseline biomarker value and *α*_3_ is the log hazard ratio for a one unit increase in the observed baseline biomarker value. Although simple to fit, Equation (6) does not account for potential measurement error in *Y*_0*i*_.

### Simulation study

In order to assess the performance of the standard approach of including observed biomarker values, compared to the full joint model described above, we evaluated both through simulation [19]. For ease of exposition we assume a longitudinal model with random intercept and slope, assuming a continuous biomarker of interest with

$$W_{i}(t_{\text{ij}})=(\beta_{0}+b_{0i})+(\beta_{1}+b_{1i})t_{\text{ij}}$$

where *β*_0_ = *β*_1_ = 0, and *b*_0*i*_ ∼ N(0,1), *b*_1*i*_ ∼ N(0,0.25^2^) with correlation between (*b*_0*i*_,*b*_1*i*_) of 0.25. Observed measurements are then generated from $Y_{\text{ij}}∼N(W_{i}(t_{\text{ij}}),\sigma_{e}^{2})$, where *t*_*ij*_ is the time of the *j*^*th*^ measurement for the *i*^*th*^ patient. We vary *σ*_*e*_ from {0.1,0.5,1}.

We assume a Weibull baseline hazard function with *λ* = 0.1 and *γ* = 1.5. A binary variable, *X*_1_ to represent treatment group was generated from Bin (1,0.5), with an associated log hazard ratio of *ϕ*_1_ = -0.5. A continuous covariate, *X*_2_, to represent age at baseline was generated from N(65,12) with an associated log hazard ratio of *ϕ*_2_ = 0.01. We then generate survival times from a Weibull distribution where the hazard is defined as *h*(*t*) = *h*_0_(*t*) exp(*α*_2_*β*_0*i*_ + *ϕ*_1_*X*_1_ + *ϕ*_2_*X*_2_), with *α*_2_ the association parameter, indicating the effect of a one unit increase in the value of the subject specific intercept on the risk of event. We vary *α*_2_ = {-0.5,0.25,0.25,0.5}. Each simulation contained 300 patients with up to 5 annual measurements (including baseline), and administrative censoring at 5 years. This corresponds to an approximate 18.9% survival proportion at 5 years (calculated at the mean of covariate values, $X_{1}=\frac{1}{2},X_{2}=65$ and *β*_0*i*_ = 0). To each dataset we fit a Weibull proportional hazards model including the observed baseline measurement, and a Weibull-based joint model with the random intercept association structure. We adjust for age and treatment in the survival submodel. Each scenario is simulated 1000 times.

**Figure 1:**
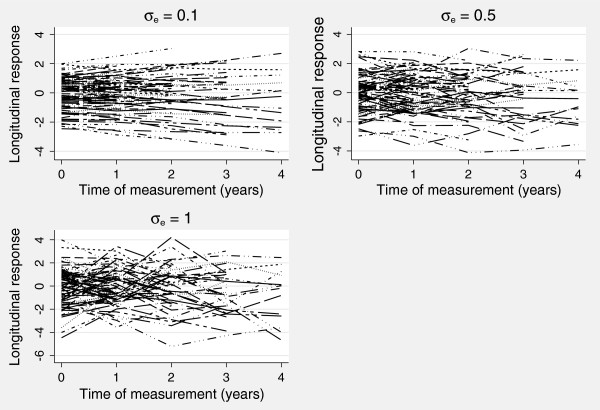
Example simulated observed longitudinal measurements with varying measurement error standard deviation.

To illustrate the varying measurement error standard deviations used in the simulation scenarios, we show in Figure 1 observed longitudinal measurements from the same 100 patients with *σ*_*e*_ = {0.1,0.5,1}, and when *α* = 0.25. Figure 1 illustrates that as the measurement error standard deviation increases, the variability in the observed biomarker values increases.

### The GPRD cohort

The General Practice Research Database (GPRD) Group has obtained ethical approval from a Multi-centre Research Ethics Committee (MREC) for all purely observational research using GPRD data; namely, studies which do not include patient involvement The core work of the GPRD is covered by MREC approval granted by the Trent Multi- Research Ethics Committee (REC reference number 05/MRE04/87) and this study was approved by the GPRD Independent Scientific Advisory Committee (ISAC) (Protocol number 09_094). This study is based in part on data from GPRD obtained under licence from the UK Medicines and Healthcare Product Regulatory Agency (MHRA). However, the interpretation and conclusions contained in this study are those of the authors alone.

The example cohort used to illustrate the methods in this paper consists of 4,850 obese patients diagnosed with type 2 diabetes mellitus. We have 107,347 measurements of SBP, with maximum follow-up of 22 years. There were 278 stroke events observed.

**Figure 2:**
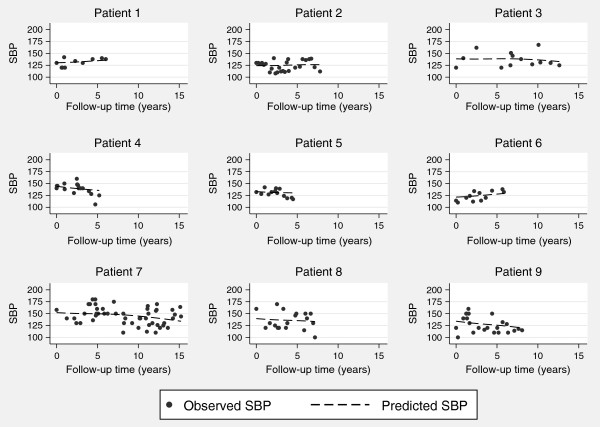
**Longitudinal response measurements for SBP for 9 randomly selected patients who had at least 10 measurements.** The dashed line represents the fitted longitudinal trajectories based on the joint model.

In Figure 2 we show the observed SBP measurements for 9 randomly selected patients, who had at least 10 measurements, illustrating some nonlinear trajectories. To accommodate such nonlinearities we can use restricted cubic splines in the linear mixed effects submodel. In particular, we specify the following longitudinal submodel

(7) $$\begin{array}{ll} \;W_{i}(t_{\text{ij}}) & =(\beta_{0}+b_{0i})+\beta_{1}{\text{age}}_{i}+\beta_{2}{\text{sex}}_{i}+\beta_{3}{\text{BMI}}_{i}\\ & \;+(\beta_{F}s_{F}(t_{\text{ij}};k_{F})+b_{R}s_{R}(t_{\text{ij}};k_{R})) \end{array}$$

Where *s*_*F*_(*t*_*ij*_;**k**_*F*_) is the restricted cubic spline basis of measurement time with corresponding fixed effects, ***β***_*F*_, with knot locations **k**_*F*_, and *s*_*R*_(*t*_*ij*_;**k**_*R*_) is the restricted cubic spline basis of measurement time with corresponding random effects, **b**_*R*_, and knot locations **k**_*R*_.

Prelimenary modelling of the longitudinal data can be conducted to guide model selection, in particular, the degrees of freedom for the spline terms capturing the underlying longitudinal trajectory over time.

To allow flexibility in the survival submodel we use the flexible parametric survival model [16,20], which models the baseline log cumulative hazard function using resticted cubic splines. We can once again undertake seperate analysis of just the survival data to inform model selection. In particular, we can use the AIC and BIC to guide the selection of the number of degrees of freedom to capture the baseline hazard function, following Rutherford et al. (2013) [21]. Our final joint model is then

(8) $$\begin{array}{ll} \;\log\left[H_{i}(t)\right] & =\log\left[H_{0}(t)\right]+\phi_{1}\;{\text{age}}_{i}+\phi_{2}\;{\text{sex}}_{i}+\phi_{3}\;{\text{BMI}}_{i}\\ & \;+\alpha_{2}(\beta_{0}+b_{0i}) \end{array}$$

where

(9) $$\log\left[H_{0}(t)\right]=s(\log(t);\gamma ,{\text{k}}_{S})$$

where the baseline log cumulative hazard function, log[*H*_0_(*t*)], is expanded into a restricted cubic spline function of log(*t*), *s*(log(*t*);*γ*,**k**_*S*_), with knot locations **k**_*S*_ and coefficient vector, *γ*. This framework has recently been incorporated into a joint model [22]. In each submodel we adjust for the baseline effects of age, sex and BMI. We fit the joint model with the random intercept association structure shown in Equation (5). For comparison, we also apply the standard flexible parametric survival model, adjusting for observed baseline SBP, age, sex and BMI.

## Results

### Simulation study results

Bias and coverage estimates for the association parameter are presented in Table 1. Under the standard Weibull model, we observe increasing bias in the estimates of the association between baseline biomarker values and survival, as the magnitude of the measurement error standard deviation, *σ*_*e*_, increases. In parallel we observe very poor coverage probabilities under the Weibull approach. For example, with *α* = 0.5 and *σ*_*e*_ = 1, we observe bias of -0.261 (percentage bias of -52.2%) and coverage of 0.4%. In contrast, under the joint modelling approach we observe minimal bias and coverage probabilities close to 95% across all scenarios.

**Table 1:** **Simulation results of the association parameter,** ***α***

**True**	**True**	**Weibull**				**Joint model**			
***α***	***σ***_***e***_	**Bias**	**% bias**	**MSE**	**CP**	**Bias**	**% bias**	**MSE**	**CP**
	0.1	-0.001	-0.2	0.006	94.8	0.005	0.9	0.006	95.3
0.50	0.5	-0.105	-21.1	0.016	65.4	0.005	0.9	0.007	95.6
	1.0	-0.261	-52.1	0.071	0.4	0.008	1.6	0.012	94.8
	0.1	0.002	1.0	0.005	94.4	0.005	2.0	0.006	94.3
0.25	0.5	-0.046	-18.5	0.007	89.0	0.007	2.7	0.007	94.5
	1.0	-0.123	-49.2	0.018	34.1	0.010	4.1	0.009	94.8
	0.1	0.003	-1.3	0.006	93.8	0.001	-0.2	0.006	94.0
-0.25	0.5	0.051	-20.6	0.007	87.1	0.000	-0.1	0.007	94.2
	1.0	0.127	-50.7	0.019	29.7	-0.002	0.9	0.009	94.6
	0.1	0.000	-0.1	0.006	96.6	-0.005	1.0	0.006	95.9
-0.50	0.5	0.104	-20.9	0.015	66.7	-0.006	1.1	0.007	95.7
	1.0	0.260	-52.0	0.070	0.4	-0.010	2.0	0.012	94.5

*MSE - mean square error*.

*CP - coverage probability*.

*σ*_*e*_*- standard deviation of the measurement error*.

### Analysis of GPRD cohort

We now present the analysis of the GPRD cohort. In all analyses we use SBP/10 so that a unit increase in SBP/10 represents a clinically meaningful 10 unit increase in SBP. Our primary interest is the association between baseline SBP and the risk of stroke. Baseline (*t*_*ij*_ = 0) corresponds to when each patient entered the cohort, i.e. the time of first SBP measurement.

We began by assuming a random intercept and selecting the degrees of freedom for the fixed spline terms using the AIC and BIC. In this case, both selected five degrees of freedom for *s*_*F*_(*t*_*ij*_;**k**_*F*_), with an AIC of 417565.8 and BIC of 417604.1. For the random splines of time we assumed a linear term, which equates to one spline term for *s*_*R*_(*t*_*ij*_;**k**_*R*_). This allows a very flexible form to take into account the variation in SBP over time. We further adjust for age, sex and Body-Mass Index (BMI) at baseline.

For the flexible parametric survival submodel, both AIC and BIC selected two degrees of freedom, with an AIC of 2408.7173 and BIC of 2430.483. If one degree of freedom had been selected, then this would be equivalent to a Weibull survival model.

Results are presented in Table 2. Under the standard flexible parametric survival model we observe a hazard ratio for a ten unit increase in baseline SBP of 1.111 (95% CI: 1.051, 1.172). Under a joint model we observe an increased hazard ratio of 1.198 (95% CI: 1.107, 1.298). The increased effect using a joint model is consistent with that observed in the simulation study, i.e. that the bias in the standard survival model is towards the null. The fitted trajectories seen in Figure 2 appear to capture the subject-specific measurements well, although some panels appear to only require a linear trend.

**Table 2:** Results from applying a flexible parametric proportional hazards model adjusting for observed baseline systolic blood pressure, and a full joint model using the intercept association structure

		**Standard FPSM**			**Joint model**		
		**Coefficient**	**95% CI**		**Coefficient**	**95% CI**	
Survival model:							
	Baseline SBP/10 (*α*_2_)	0.105	0.050	0.159	0.181	0.102	0.261
	Age (years)	0.048	0.036	0.060	0.050	0.038	0.062
	Sex (male)	0.011	-0.233	0.254	-0.010	-0.253	0.234
	BMI (kg/m^2^)	0.011	-0.015	0.037	0.013	-0.012	0.039
Longitudinal model:							
	Intercept	-	-	-	13.006	12.629	13.382
	Age (years)	-	-	-	0.025	0.022	0.029
	Sex (male)	-	-	-	-0.252	-0.332	-0.171
	BMI (kg/m^2^)	-	-	-	0.003	-0.005	0.011
	RCS1	-	-	-	-0.080	-0.121	-0.039
	RCS2	-	-	-	-0.006	-0.019	0.006
	RCS3	-	-	-	-0.001	-0.010	0.007
	RCS4	-	-	-	0.003	0.000	0.006
	RCS5	-	-	-	0.000	-0.001	0.001
	*σ*_*e*_	-	-	-	1.522	1.515	1.528

*FPSM - Flexible Parametric Survival Model*.

*RCS - Restricted Cubic Spline*.

**Figure 3:**
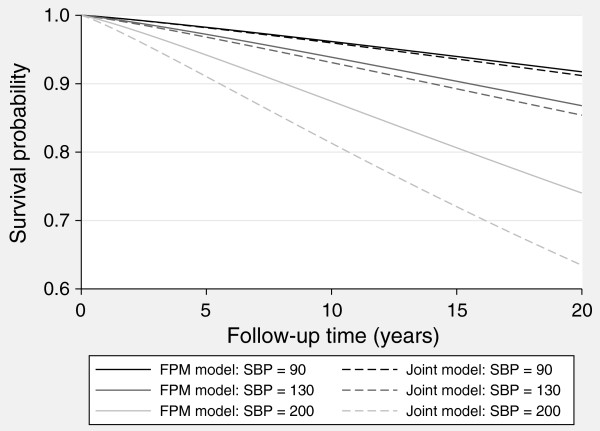
Predicted survival from the flexible parametric survival model and joint model, for a female, aged 60 years, BMI of 30, with SBP of 90, 130 or 200.

We illustrate how the bias from the standard approach increases with SBP in Figure 3, showing predictions from both models for a female patient aged 60, with low (90), medium (130) and high (200) SBP baseline measurements. To quantify the differences, at 10 years under the standard model we observe a survival probability of 0.881 for a SBP of 200, compared to 0.816 under the full joint model.

## Discussion

A wealth of patient data is becoming available in registry sources such as the GPRD, providing extensive opportunities to utilise the joint modelling framework. We have shown that by incorporating repeated measures of a biomarker within a unified joint model framework, we reduce bias due to measurement error, even when only the baseline level of the biomarker is predictive of survival. As illustrated in the simulation study, ignoring measurement error in biomarkers such as blood pressure can lead to a marked underestimation of covariate effects. In our application, through the use of restricted cubic splines in the linear mixed effects submodel, we can model highly nonlinear trajectories over time, compared to linear slope models. Furthermore, the flexible parametric survival submodel can also capture complex baseline hazard functions, an important component when predicting survival at the patient level [22].

Given that, to our knowledge, all current cardiovascular risk scores only use baseline measures, with no adjustment for measurement error, the prospects of utilising this framework to improve prognostic risk scores is quite substantial. Predicting survival for a new patient using this framework follows naturally, as often only a first baseline biomarker observation will be available. However, such a modelling approach also allows a dynamic risk prediction approach to be adopted, whereby a patient’s estimated future risk is updated as each new biomarker value is obtained [23]. Such an approach could enable response to treatment to be monitored and patients counselled accordingly.

In the analysis of the GPRD cohort, we incorporated flexibility in both the longitudinal submodel through the use of restricted cubic splines, and the flexible parametric survival submodel. Given that both submodels require choosing the number of degrees of freedom, a simple sensitivity analysis can be undertaken to assess knot locations and number of knots. We showed recently that the flexible parametric survival submodel is very robust to both knot placement and number of knots within a joint model framework [22], and furthermore, an extensive simulation study has been conducted by Rutherford et al. (2013), which showed excellent performance of the flexible parametric model to capture simple and complex baseline hazard functions [21]. Furthermore, given that primary interest was in the survival component, and the estimate of association, often modelling the longitudinal component with a suitable sensible functional form will provide an improved estimate compared to simplistic approaches of seperate modelling.

In this paper we have concentrated on a specific association structure linking the 2 component submodels; however, it may be of interest to investigate linking multiple components of a biomarkers trajectory to the time to an event of interest. For example, recent work by Rothwell et al. (2010) [24] has shown associations between not only baseline blood pressure, but also variability over time as important predictors of cardiovascular events. Furthermore, we have only compared the standard approach of adjusting for observed baseline biomarker values to the full joint model. It would be of interest to compare alternative approaches for adjusting for measurement error, not only in baseline biomarkers, but also under a time-dependent association structure [25,26].

Extensions to the modelling framework include incorporating multiple biomarkers. In particular, in our example we modelled SBP over time, whilst adjusting for baseline BMI. It may be of interest to model not only SBP but also the inter-relationships between different biomarkers such as BMI, and how they are related to an event of interest [13].

To facilitate the use of the methods in practice, user friendly Stata software, written by the first author, is available, with a variety of survival model choices and association structures, including those discussed in this article [9,27]. To illustrate computational aspects of the framework, the presented joint model applied to the cohort took just over 13 minutes to converge on a standard laptop computer.

## Conclusion

The joint modelling of longitudinal and survival data is a valid approach to account for measurement error in the analysis of a repeatedly measured biomarker and a time to event. User friendly Stata software is provided.

## Competing interests

The authors declare that they have no competing interests.

## Authors’ contributions

All authors were involved in conception and design of the project. MJC conducted the simulation study, analysed the clinical dataset and wrote the first draft of the manscript. PCL and KRA both revised the manuscript. All authors read and approved the final manuscript.

## Pre-publication history

The pre-publication history for this paper can be accessed here:

http://www.biomedcentral.com/1471-2288/13/146/prepub
